# Microvascular rarefaction caused by the NOTCH signaling pathway is a key cause of TKI-apatinib-induced hypertension and cardiac damage

**DOI:** 10.3389/fphar.2024.1346905

**Published:** 2024-02-09

**Authors:** WenJuan Wang, Guodong Li, Jie Ma, Xin Fan, Jianzhong Lu, Qiyin Sun, Jiafang Yao, Qingjian He

**Affiliations:** ^1^ Department of Cardiovascular Center, The First People’s Hospital of Huzhou City, Huzhou, China; ^2^ Department of Hypertension Center, Lanzhou University Second Hospital, Lanzhou, China; ^3^ Department of Breast and Thyroid Surgery, The First People’s Hospital of Huzhou City, Huzhou, China

**Keywords:** hypertension, microcirculation, rarefaction, TKIs, apatinib

## Abstract

With the advancement of tumour-targeted therapy technology, the survival of cancer patients has continued to increase, and cardiovascular events have gradually become an important cause of death in cancer patients. This phenomenon occurs due to adverse cardiovascular reactions caused by the cardiovascular toxicity of antitumour therapy. Moreover, the increase in the proportion of elderly patients with cancer and cardiovascular diseases is due to the extension of life expectancy. Hypertension is the most common cardiovascular side effect of small molecule tyrosine kinase inhibitors (TKIs). The increase in blood pressure induced by TKIs and subsequent cardiovascular complications and events affect the survival and quality of life of patients and partly offset the benefits of antitumour therapy. Many studies have confirmed that in the pathogenesis of hypertension, arterioles and capillary thinness are involved in its occurrence and development. Our previous findings showing that apatinib causes microcirculation rarefaction of the superior mesenteric artery and impaired microvascular growth may inspire new therapeutic strategies for treating hypertension. Thus, by restoring microvascular development and branching patterns, total peripheral resistance and blood pressure are reduced. Therefore, exploring the key molecular targets of TKIs that inhibit the expression of angiogenic factors and elucidating the specific molecular mechanism involved are key scientific avenues for effectively promoting endothelial cell angiogenesis and achieving accurate repair of microcirculation injury in hypertension patients.

## 1 Introduction

Hypertension has a high prevalence in China and accounts for a large proportion of cardiovascular diseases. Hypertension is also a major risk factor for cardiovascular-related diseases. In recent years, a number of studies have reported a relationship between tumours and hypertension. Tumours and hypertension have common risk factors and overlapping pathophysiological mechanisms (Milan et al., 2014; Bray et al., 2021; Dolmatova et al., 2023). Therefore, several experts have gradually formed the theory of onco-hypertension. Previous studies by our group have shown that hypertension and antihypertensive drug use are closely related to breast cancer. With increasing age, the incidence of hypertension in breast cancer patients increases, and the mechanism is related to inflammatory mediators and angiogenesis (Zhao et al., 2018; Wang et al., 2021). Another reason is that women’s estrogen levels decline as they age. Estrogen has a protective effect on blood vessels, so such patients are more likely to induce hypertension. And estrogen for breast cancer patients, with the increase of age, although estrogen in the body decreases, but some patients may be converted to androgen, leading to the increase in the incidence of breast cancer. In recent years, new antineoplastic drugs have prolonged the survival of cancer patients, but the increase in blood pressure caused by new antineoplastic drugs and the subsequent cardiovascular complications and events affect the survival and quality of life of patients, which partly offset the benefits of antineoplastic therapy. It is worth exploring this topic further.

## 2 Incidence of hypertension caused by TKIs

VEGF signalling pathway inhibitors include monoclonal antibodies against VEGF A factor, VEGF traps, monoclonal antibodies against VEGF receptors, and TKIs (Pucci et al., 2019; Le et al., 2021; Lawler, 2022). Studies have shown that the probability of hypertension caused by VEGF signalling pathway inhibitors during antitumour therapy is approximately 11%–45%, among which the incidence of hypertension above grade 3 (referring to CTCAE grade 3–4, that is, SBP≥160 and/or DBP ≥100 mmHg) or life-threatening hypertension, even requiring emergency treatment, ranges from 2 to 20% (Xu et al., 2021). According to the type and dose of TKIs, the incidence of hypertension can reach 20%–90%, and the incidence of severe hypertension can reach 6%–43% in patients using TKIs alone or in combination. The incidence of hypertension induced by TKIs is shown in Table 1.

**TABLE 1:** Incidence of hypertension caused by TKIs.

Drug	Category	Target spot	Hypertension (%)
axitinib	TKIs	VEGFR1-3,c-KIT,PDGFR	22–84
cabozantinib	TKIs	MET,VEGFR2,RET,AXL,FLT3	28–61
Lenvatinib	TKIs	VEGFR1-3, FGFR 1–4,PDGFR, c-KIT,RET	42–73
Pazopanib	TKIs	VEGFR1-3,PDGFR, FGFR,c-KIT	40–42
Ponatinib	TKIs	BCR-ABL,VEGFR, PDGFR,FGFR,EPH,c-KIT,RET,TIE2,FLT3	53–74
regorafenib	TKIs	VEGFR1-3,PDGFR,c-KIT,RET,RAF-1	28–67
Sorafenib	TKIs	VEGFR1-3,PDGFR,c-KIT,RET,RAF-1	4–31
sunitinib	TKIs	VEGFR2,PDGFR,c-KIT	20–27
Wandtaneb	TKIs	VEGFR2,PDGFR,c-KIT	4–40

Apatinib mesylate is an antitumour drug that was independently developed in China and belongs to the class of TKIs. In the past, it was mainly used for the treatment of solid tumours, such as advanced gastric cancer or gastroesophageal junction adenocarcinoma (Li et al., 2016). An international multicentre phase III study (SHR-1210-III.-310 study) demonstrated that the approved PD-1 inhibitor camrelizumab in combination with apatinib had a significant survival benefit and a tolerable safety profile in the first-line treatment of advanced liver cancer, with a median overall survival (OS) of 22.1 months. It is the combination therapy with the longest OS benefit in the first-line treatment of advanced liver cancer (Qin et al., 2023). Therefore, the National Medical Products Administration (NMPA) proposed the combination of “Shuang’ai" for the first-line treatment of advanced hepatocellular carcinoma, which is the first approval in China to use a combination of PD-1 inhibitors and TKIs for the treatment of advanced hepatocellular carcinoma. The application of this treatment regimen has led to new drug options for patients with advanced liver cancer. The resulting cardiovascular side effects of nitrogen are also very noteworthy. Therefore, studying the cardiovascular toxicity and side effects caused by TKIs is highly important for guiding antitumour therapy in clinical cancer patients.

## 3 Causal relationship between microcirculation damage and hypertension

### 3.1 Microcirculation and related definitions of microcirculation damage

Microcirculation typically includes small resistance arteries (300–100 µm in diameter), precapillary arterioles (100–10 µm), capillaries (5–15 µm) and venules (10–100 µm) (le Noble et al., 2023). Various studies have shown that damage to microcirculatory tissue may involve multiple mechanisms. The main categories of such damage include impaired endothelial cell function, oxidative stress, decreased angiogenesis (most commonly in patients after the use of targeted drugs), increased endothelial permeability, enhanced leukocyte adhesion, immune cell activation, lymphatic dysfunction, impaired autoregulation, microvascular constriction, and microcirculation obstruction (Sorop et al., 2020; Sabe et al., 2022; De Ciuceis et al., 2023; Mengozzi et al., 2023).

### 3.2 Microcirculation and types of microcirculation damage

Studies have shown a relationship between hypertension and changes in the microvascular network in spontaneously hypertensive rats (SHRs), with reduced arteriolar density and increased postcapillary venule density (Martens and Gelband, 1998). It lead to increased postvascular resistance, which may further contribute to the development of hypertension. In addition, it has been shown that a sparse microvascular network structure increases total peripheral resistance, which eventually leads to increased blood pressure (le Noble et al., 2023). Many previous studies have confirmed the importance of arteriolar and capillary scarcity in the pathogenesis of hypertension (Levy et al., 2001; Levy et al., 2008). That is, the rarefaction of capillaries may contribute to the development of hypertension.

Microcirculation injury can occur through two main forms of microvascular rarefaction (Agabiti-Rosei, 2003; Ungvari et al., 2021). Functional rarefaction means that the total number of anatomically present vessels is not reduced but rather that perfusion of this part of the microvascular network is absent. However, as the vascular tone continues to increase, the lumen area increases, resulting in a decrease in the number of blood vessels perfused. Structural sparseness also occurs, which reduces the number of blood vessels that can be found during tissue dissection. This reduction in vascular mesh may be due to altered anatomical changes in the vascular segments or other impairments in the vascular network during the growth and development process during early tissue development. Several experts have also noted that functional rarefaction can progress to structural rarefaction in a rat model of SHR. However, there are also studies showing that patients with essential hypertension, recruitment of perfused capillaries is impaired, which can be explained by both functional and structural rarefaction (Serné et al., 2001). Therefore, whether the microcirculation dilution in hypertension is structurally sparse or functional, or even both, will be our further research plan. This part of the study is crucial for them to identify the specific microcirculation sparse so that we can improve and treat it according to the cause, especially in patients with hypertension caused by targeted drugs.

### 3.3 Mechanisms of microcirculation damage leading to hypertension

Microvascular injury plays an indispensable role in the occurrence and development of hypertension, and some studies have shown that it plays an important role in the progression of hypertension, hypertension-mediated organ damage and related cardiovascular events (Jonk et al., 2007; Karaca et al., 2014; Rizzoni et al., 2023). However, the causal relationship between thinning of microvessels and hypertension is difficult to explain, and some experts speculate that diffuse generalized thinness of microvessels may be one of the main causes of hypertension (Marinescu et al., 2015; Horton and Barrett, 2021). In study, after 5 weeks of treatment with telatinib, the capillary density decreased from 20.8 at baseline to 16.7 (Steeghs et al., 2008). Mourad et al. reported a significant reduction in kinetic and structural capillary density in a group of patients with metastatic colon cancer treated with bevacizumab for 6 months (Mourad et al., 2008). However, this rarefaction is most likely functional because blood pressure increases rapidly after the start of treatment and returns to normal immediately after the discontinuation of VEGF inhibitors (van Dorst et al., 2021). Therefore, further studies to determine whether microvascular thinning is the cause or effect of the actions of VEGF inhibitors on hypertension are highly clinically important.

Animal experiments revealed that a rise in blood pressure leads to an increase in the production of reactive oxygen species in mice. Therefore, researchers have speculated that elevated blood pressure may be responsible for microvascular function and structural alterations, further contributing to the manifestation of vascular thinning (Ungvari et al., 2004; Jacobson et al., 2007). However, there is considerable evidence that microvascular changes may also be a cause rather than a consequence of hypertension. In animal models of hypertension, increased reactive oxygen species production and sparse arterioles occur even in the vasculature not exposed to hypertension (Boegehold et al., 1991). Our previous results showed that blood pressure was significantly increased in an apatinib-treated mouse model of gastric cancer, and sparsity of arteriolar vessels was detected in the mesenteric arteries of experimental model mice. Therefore, we speculate that the microcirculation damage caused by apatinib during antitumour treatment may be one of the main causes of hypertension. However, this study did not further confirm whether functional or structural vascular alterations are the cause, and it is very important to clarify this classification for the treatment of hypertension induced by TKIs. Because we did not further lower the blood pressure after the elevation of blood pressure, we investigated whether the vascular density of the superior mesenteric artery in the mice was further restored.

In addition, microvascular rarefaction is observed in the early stages of hypertension development, and microvascular rarefaction can be detected in individuals with a family history of hypertension, even if their blood pressure is normal. Similarly, in animal experiments, microvascular abnormalities occur early in the development of hypertension in SHRs (Antonios et al., 2003). In addition, the latest results from our research indicated that the administration of the ROCK inhibitor Y27632 can prevent microvascular rarefaction caused by apatinib and improve blood pressure (Wang et al., 2022a). Thus, microvascular growth disorders may have given rise to a new paradigm of hypertension treatment aimed at restoring microvascular development and branching, thereby further reducing peripheral resistance and improving the structural reduction of arterial blood pressure.

## 4 Relationship between microcirculation-induced hypertension and heart failure

Hypertension is an important risk factor for heart failure (Garg et al., 2021). Long-term uncontrolled hypertension can exert excessive pressure on the heart and gradually impair heart function, leading to cardiac hypertrophy and decreased myocardial contractility. Over time, this overload can impede the ability of the heart to pump blood effectively, triggering heart failure (Slivnick and Lampert, 2019; Jackson et al., 2021; Redfield and Borlaug, 2023). The most common type of heart failure is heart failure with preserved ejection fraction (HFpEF). There are multiple mechanisms involved in the development of hypertension from HFpEF, and these mechanisms can be divided into several categories. The most common is microcirculatory dysfunction, which mainly includes an increase in inflammatory factors, the occurrence of oxidative stress, impaired endothelial function, and the occurrence of vascular endothelial fibrosis (Gryglewska et al., 2011; Paulus and Tschöpe, 2013; Stupin et al., 2021; Marra et al., 2022).

Firstly, left atrial pressure increases in hypertensive patients, with a consequent increase in left ventricular end-diastolic pressure, and left atrial remodeling occurs. This process is an indication of left ventricular diastolic dysfunction. Left atrial enlargement and dysfunction, including reduced left atrial systolic reserve, can eventually lead to the development of HFpEF (Nagai et al., 2023; Li et al., 2024). Secondly, in patients with chronic systemic inflammation such as hyperemia, systemic secreted inflammatory cytokines can cause the accumulation and inflammatory response of epicardial adipose tissue, which can promote the migration and transformation of mesenchymal stem cells and local secretion of inflammatory cytokines, leading to deep myocardial cell inflammation, increased myocardial stiffness, deep myocardial fibrosis, and finally HFpEF (Obokata et al., 2017; Gevaert et al., 2022; Gao et al., 2023; Rossi et al., 2023). In addition, microcirculation disorders also play an important role in the occurrence and development of HFpEF (Camici et al., 2020). Systemic inflammation can lead to microvascular inflammation and endothelial activation, resulting in significant structural and functional changes in cardiomyocytes and extracellular matrix, including the decrease of NO and vasodilator peptide levels, which leads to the enhancement of vasoconstriction, left ventricular stiffness and myocardial collagen-ization, and ultimately the formation of HFpEF (Vancheri et al., 2020; Agrawal et al., 2023). Overall, in most patients with hypertension, left ventricular diastolic dysfunction is the first apparent manifestation of heart failure. Our previous study (Wang et al., 2022a) showed that apatinib increased blood pressure in gastric cancer model mice, which further led to left ventricular hypertrophy and fibrosis. Therefore, exploring the mechanism through which apatinib-induced hypertension leads to HFpEF in gastric cancer mice is highly important, as is exploring drugs that can delay the progression of HFpEF.

Abnormal structure and function of miniscule arteries and capillaries results in hypoperfusion of blood flow (Mourad and Levy, 2011; Struijker-Boudier and Heijnen, 2011; Wu et al., 2023). Studies have shown that sparse microcirculation may be one of the main causes of hypertension (Ciuffetti et al., 2003). Chronic high blood pressure damages the heart and blood vessels. Pathological changes in miniscule arteries and capillaries, such as intimal thickening, fibrosis, and luminal narrowing, further inhibit blood flow perfusion (Triantafyllou et al., 2015). This microcirculatory pathology affects normal oxygen availability and nutrient delivery to the heart and other tissues (De Backer et al., 2014; Cusack et al., 2022). Ischaemia and hypoxia lead to cardiac cell damage and death, which eventually leads to weakened cardiac systolic function and the occurrence of heart failure (Premont et al., 2020). Therefore, microcirculatory impairment plays a key role in hypertension and cardiac dysfunction. Therefore, protecting microcirculation function is highly important for the prevention and treatment of hypertensive heart failure.

## 5 Mechanisms related to hypertension caused by TKIs

### 5.1 Relationship between VEGF signaling pathway and TKI-induced hypertension

The most common pathways are the vascular endothelial growth factor (VEGF), platelet-derived growth factor (PDGF), angiopoietin-1, and Notch signalling pathways (Viallard and Larrivée, 2017; Vimalraj, 2022; Huang et al., 2023) and so on. VEGF is one of the most important proangiogenic mediators. Recently, studies have shown that VEGF is the core factor affecting endothelial cell angiogenesis and is closely related to hypertensive microcirculation damage (Chade and Kelsen, 2010). However, the foundation of TKI antitumour therapy is the inhibition of vascular endothelial growth factor, which brings certain challenges to antitumour therapy. The latest research from our team revealed that in a mouse model of gastric cancer, the mechanism of apatinib-induced hypertension in mice may be related to the sparse vascular density of the superior mesenteric artery (Wang et al., 2022a).

VEGF signaling pathway inhibitors include monoclonal antibodies against VEGFA, vascular endothelial growth factor inhibitors (VEGF trap), monoclonal antibodies against VEGF receptors, and TKIs (Saif, 2013; Shughoury et al., 2023). TKIs are effective signaling cascade inhibitors that inhibit tumor blood vessel growth by inhibiting vascular endothelial growth factor receptor (VEGFR) (Vano et al., 2022). VEGFR-TKIs have become the main treatment for many solid malignant tumors. However, TKIs can induce vascular endothelial damage, hypertension and myocardial injury by targeting VEGFR, platelet-derived growth factor receptor (PDGFR) and stem cell factor receptor (SCFR) (van Cruijsen et al., 2009; Lennartsson and Rönnstrand, 2012). It can also damage mitochondria and affect myocardial energy metabolism through the “off-target effect", eventually leading to cardiovascular complications (Tullemans et al., 2018; Rodríguez-Agustín et al., 2023). Therefore, the incidence of cardiovascular toxicity related to VEGFR-TKIs is high, which can cause the occurrence and development of cardiovascular complications such as hypertension, left ventricular systolic dysfunction/heart failure, and atherosclerosis.

Anti-angiogenesis targeting drugs mainly act on VEGF and VEGFR. VEGFR-1, VEGFR-2 and VEGFR-3 are the major VEGF receptors (Sallinen et al., 2009; Bernatz et al., 2021). VEGF inhibitors can increase the risk of heart failure, coronary heart disease, hypertension and thromboembolic diseases through endothelial injury, vasoconstriction and remodeling, inflammatory response and platelet activation.

### 5.2 Relationship between RhoA/ROCK signaling pathway and TKIs-induced hypertension

In addition, the latest study from our team revealed that apatinib has a considerable therapeutic effect on a mouse model of gastric cancer (Wang et al., 2022a). However, apatinib can also lead to an increase in blood pressure, accompanied by activation of the RhoA/ROCK signalling pathway. And the ratio between vessel thickness and lumen diameter was significantly increased in the apatinib group. We also observed that apatinib in combination with the ROCK inhibitor Y27632 did not affect the antitumour therapeutic effect of apatinib. Finally, our combined administration of ROCK inhibitors significantly reduced the increase in blood pressure in an apatinib-induced gastric cancer mouse model (Wang et al., 2022a). Therefore, Y27632 has wide application prospects for the treatment of tumour-induced hypertension.

Further studies at the cellular level have shown that knocking down the key gene LRAG in the RhoA/ROCK signalling pathway can improve the abnormal proliferation and impaired cell function of vascular smooth muscle cells caused by apatinib (Wang et al., 2022b). This is mainly because apatinib can increase blood pressure by activating the RhoA/ROCK signaling pathway. Therefore, exploring the key molecular targets of TKIs that inhibit the expression of angiogenic factors and elucidating the specific molecular mechanism involved are key for effectively promoting endothelial cell angiogenesis and achieving accurate repair of microcirculation injury in hypertension patients.

### 5.3 Relationship between notch signaling pathway and TKIs-induced hypertension

Notch and DLL4 are specifically expressed on vascular endothelial cells (EC). JAG has been shown to promote cell survival and proliferation, interacting with NOTCH and hematopoietic stem and progenitor cells (HSPCs) (Kangsamaksin et al., 2015). In addition, high expression of JAG promotes cancer development. We hypothesized that inhibition of Notch signaling activation would not only inhibit tumor development, but also help angiogenesis. Therefore, exploring the mechanism of angiogenesis dysfunction induced during antitumour therapy is highly clinically important.

Notch signalling pathway, as a classical signaling pathway, is closely related to cardiovascular and tumor microcirculation. Combined with our previous results, Notch signaling pathway may also be involved in TKI-induced blood pressure elevation. The regulation of the Notch signalling pathway is closely related to the proliferation, migration and tube formation of vascular endothelial cells during tumour angiogenesis. Further research on the relationship between the Notch signalling pathway and tumour-related cardiovascular disease is expected to lead to new therapeutic strategies and targets for the prevention and treatment of tumours.

### 5.4 Other mechanisms associated with TKIs-induced hypertension

At present, there are many studies on hypertension caused by TKIs, and the relevant mechanisms involved may be related to the following aspects: 1) decreased bioavailability of nitric oxide (Robinson et al., 2010); 2) enhanced oxidative stress (Neves et al., 2018); 3) increased secretion of endothelin-1 (Mirabito Colafella et al., 2020); 4) decreased bioavailability of prostacyclin (Wheeler-Jones et al., 1997); 5) increased bioavailability of endothelial microparticles (Neves et al., 2019); 6) sparse microvessels (Steeghs et al., 2008); 7) vascular sclerosis (Catino et al., 2018); 8) activation of renin angiotensin (Li et al., 2022); and 9) salt-sensitive hypertension (Tsai et al., 2017).

## 6 Role of the notch signalling pathway in the cardiovascular system

**FIGURE 1:**
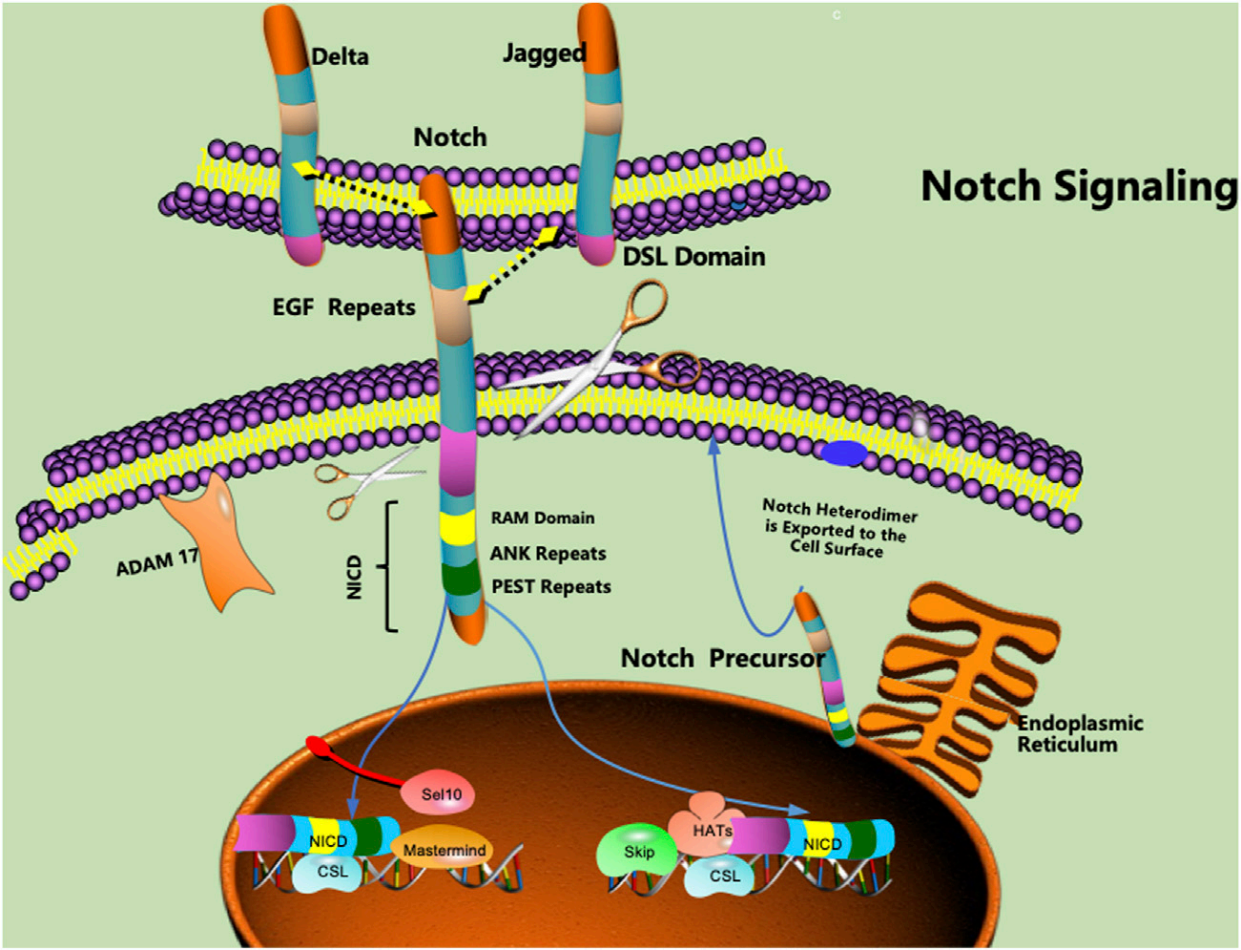
Notch proteins are cell surface transmembrane-spanning receptors that mediate critically important cellular functions through direct cell‒cell contact. EGF: epidermal growth factor, NICD: intracellular domain of Notch, NLS: nuclear localization signal, CSL: CBF1/Su(H)/Lag-1.

The Notch signalling pathway is a common signalling pathway that plays a certain role in cell development. The Notch protein is a transmembrane receptor that is located on the cell surface and mediates important cellular functions (Kwak et al., 2022; Takahashi et al., 2023). The interaction between the Notch protein and its ligand initiates a signalling cascade associated with cell fate that plays a key role in differentiation, proliferation, and apoptosis in many tissue types (Zhao et al., 2019; Li et al., 2020). The Notch protein, as well as its ligand, contains extracellular EGF-like repeats that interact with the DSL domain of the ligand. Activation of the Notch signalling pathway is accompanied by proteolysis, which releases the intracellular domain of Notch (NICD) (Sprinzak and Blacklow, 2021; Fang et al., 2022). The NICD is a fragment containing a RAM23 domain (RAM) that can enhance interactions with the Notch and CSL proteins, thereby facilitating the transmission of Notch to the nuclear localization signal (NLS) (Lubman et al., 2007; Johnson and Barrick, 2012). The NICD fragment can further mediate interactions with other proteins in the Notch signalling pathway (Figure 1).

The VEGF signalling pathway interacts with the Notch signalling pathway, coordinates the differentiation of arteries and veins, and is also involved in changes in vascular budding and vascular branching (Laham et al., 2003; Li et al., 2017; Souilhol et al., 2022; Li et al., 2023a). In addition, the VEGF signalling pathway can regulate key processes such as lumen remodelling of blood vessels.

Microvascular rarefaction leading to hypertension involves multiple signalling pathways, such as vascular endothelial development, angiogenesis, inflammation, oxidative stress, and endogenous short peptides (Feihl et al., 2008; Bruno et al., 2018; Do et al., 2023; Zdravkovic et al., 2023). Vascular endothelial cells play a key role in maintaining normal vascular biological function and regulating vascular tone. The abnormal regulation of vascular endothelial growth factor and platelet-derived growth factor during angiogenesis may also be involved in the occurrence of microvascular rarefaction (Cohen et al., 2023). Studies have shown that the Notch signalling pathway plays an important role in angiogenesis (Maynard et al., 2003).

The Notch signalling pathway plays a key role in the regulation of angiogenesis, especially in balancing the proangiogenic effects of the VEGF signalling pathway. The Notch signalling pathway antagonizes angiogenesis *via* VEGF signalling. It also stimulates quiescent endothelial cells and promotes apical cell formation. Thus, this pathway mediates the formation of vascular buds and the further growth of new vascular buds (Marinescu et al., 2015). Under conditions of stress, the cascade between Dll4-mediated Notch signalling and VEGFA-VEGFR2 signalling induces endothelial cells adjacent to the dominant TCS to maintain high levels of Notch signalling, thereby inhibiting their differentiation into TCShh cells. Furthermore, it can inhibit angiogenesis (Gerhardt et al., 2003; Jakobsson et al., 2010; Herbert and Stainier, 2011). Angiogenesis inhibition is thought to be controlled by Notch 1-mediated downregulation of Flt 4 and upregulation of soluble Flt 1 (sVEGFR-1/sFlt 1) (Chappell et al., 2009; Trindade et al., 2012). In particular, upregulation of sFlt1 reduces local VEGF bioavailability and inhibits angiogenesis. The Notch ligand Jag 1 is expressed mainly in stem cells and is thought to specifically block Dll 4-Notch 1 signalling in apical cells (Benedito et al., 2009). In the cardiovascular system, excessive activation of the Notch signalling pathway can lead to vascular endothelial cell proliferation and congestion and an increase in vessel wall thickness, which leads to vascular stenosis and hypertension (Niessen and Karsan, 2007; MacGrogan et al., 2018; Gomez et al., 2021).

The Notch signalling pathway is also involved in the occurrence and development of heart failure. Activation of Notch signalling can lead to cardiomyocyte proliferation and hypertrophy and can also affect angiogenesis and repair processes in the cardiovascular system (Fortini et al., 2014; Matsushita et al., 2023). These changes may adversely affect myocardial function and lead to the development of heart failure. Notch1 is a transmembrane receptor found in a variety of cells, including smooth muscle cells and endothelial cells in the cardiovascular system. During heart wall formation, Notch signalling regulates the ratio of cardiomyocytes to noncardiomyocytes by inhibiting myogenesis, thereby further promoting atrioventricular canal remodelling and maturation and heart valve formation (Niessen et al., 2011; Peng et al., 2023). Activation of the Notch1 signalling pathway is achieved mainly through the binding of the Notch1 receptor to its ligand. When the Notch1 receptor binds to its ligand, secondary cleavage occurs, and the active region of the Notch1 receptor is released. Then the receptor enters the nucleus and interacts with transcription factors to promote gene transcription and expression (Wang S. et al., 2022). Studies have also shown that activation of the Notch1 signalling pathway is closely related to cardiac function and can affect the proliferation and differentiation of cardiomyocytes, thus affecting the development and function of the heart (Pahlavani, 2022). In addition, activation of the Notch1 signalling pathway can also regulate the expression of the actin gene in cardiac cells, affecting cardiac contractility and cardiac contraction rhythm (Hrstka et al., 2017). Therefore, the Notch signalling pathway plays an integral and critical role in maintaining and regulating blood pressure and cardiac function.

## 7 Role of the notch1 signalling pathway in tumour systems

The Notch signalling pathway is involved in cell-to-cell interactions and communication and plays an important role in embryonic development and the maintenance of tissue homeostasis in adult organisms. Moreover, aberrant Notch signalling pathway activity has also been found to be closely related to the occurrence and development of a variety of tumour types.

In terms of tumours, studies have shown that abnormal activation of the Notch1 signalling pathway is related to the occurrence and development of a variety of diseases, such as tumour proliferation and metastasis and abnormal responses of the immune system (Yang et al., 2018; Peng et al., 2023). Therefore, we hypothesized that inhibition of the Notch1 signalling pathway may ameliorate the adverse effects on blood pressure and cardiac function. Therefore, what is the underlying mechanism involved in reducing blood pressure and cardiac dysfunction? This will be another important dimension that needs to be studied.

In hepatocellular carcinoma (HCC), Notch signalling is activated by different ligands and plays a polymorphic role depending on the cell type, affecting tumour growth, invasive ability, and stem cell-like properties (Giovannini et al., 2021). Thus, interfering with Notch signalling may be a promising therapeutic approach. In tumour therapy, the downregulation of Notch1 can achieve synergistic effects and reduce chemoresistance when targeted drugs are used alone or in combination with chemotherapy. In addition, Notch mutations have been proposed to be predictive biomarkers for immune checkpoint blockade therapy in many cancers (Zhou et al., 2022; Li et al., 2023b).

## 8 Possible treatment of hypertension and myocardial damage caused by the notch signalling pathway

Recently, it has been observed that Epac1 can negatively regulate the Notch signalling pathway. Epac1 knockdown in endothelial cell lines resulted in a significant increase in the intracellular Notch1 protein level, and Epac1 knockdown increased the protein levels of NICD and DLL4, thereby further inhibiting angiogenesis. Therefore, overexpression of Epac1 may be an effective way to alleviate microvascular rarefaction (Fritz et al., 2015; Lan et al., 2020; Slika et al., 2023). Interestingly, studies have shown that Epac1 knockdown can inhibit pathological angiogenesis but has no significant effect on physiological angiogenesis, which is worthy of attention and discussion (Lan et al., 2020). Previous studies have shown that Epac1 is a β-secretase enzyme that inhibits the activation of the Notch signalling pathway (Fujita et al., 2017; Tan et al., 2022). In addition, Epac1 has been shown to enhance the VEGF signalling pathway and promote pathological angiogenesis (Namkoong et al., 2009; Ramos et al., 2018). Therefore, we speculate that the occurrence of hypertension caused by TKIs may lead to microcirculation disorders and microvascular thinning, and overexpressing the Epac1 gene may be a very meaningful way to improve or treat microvascular thinning caused by TKIs. NOTCH mutations can be used as predictive markers for treatment with immune checkpoint blockade in some tumors. In summary, it is necessary to comprehensively evaluate the NOTCH pathway from a new perspective, so as to apply it in the subsequent clinical diagnosis and treatment.

## 9 Mitochondrial dysfunction may also be involved in the regulation of notch signalling

In addition, Epac1 can play a role in cell-to-cell molecular conduction by increasing its interaction with macromolecular complexes, including voltage-dependent anion channel 1 (VDAC1), chaperone glucose-regulated protein 75 (GRP75), and inositol triphosphate receptor 1 (IP3R1). The interaction between Epac1 and macromolecular complexes can further promote the exchange of Ca2+ between the endoplasmic reticulum and mitochondria, which can eventually lead to mitochondrial Ca2+ overload and the opening of mitochondrial permeability transition pores (Fazal et al., 2017). Therefore, Epac1 is expected to be a new target for the treatment of ischaemic myocardial injury.

Mitochondria are important organelles for cellular energy production, and dysfunction of these organelles plays a key role in the emergence and progression of cardiovascular diseases (Poznyak et al., 2021; Wu et al., 2022). Normally, mitochondrial proteostasis is monitored by an extensive system: the mitochondrial unfolded protein response (UPRmt) is activated when mitochondria are stimulated by misfolded protein stress. The latter promotes the upregulation of ClpP, HSP6, HSP-60, ATFS-1 and other markers through a series of signalling cascades to alleviate mitochondrial stress (Merkwirth et al., 2016; Kumar et al., 2022; Xin et al., 2022; Guo et al., 2023). Under conditions of stress, cells can protect mitochondria by activating the UPRmt. It has been shown that the inhibition of Epac1 prevents ferroptosis-induced cell death and disruption of mitochondrial integrity, whereas the inhibition of Epac2 has a limited effect (Laudette et al., 2021; Musheshe et al., 2022). However, the potential effects of Epac on mitochondrial function are still unclear, and additional experiments are needed for further elucidation.

Several studies have shown that UPRmt activation is accompanied by the upregulation of the mitophagy markers PINK1, PARK2, BNIP3, P62, and LC3 and the mitochondrial oxidative phosphorylation (OXPHOS) markers Cox5a, Cox2, Nd1, and Sdhc. These results indicate that the UPRmt, mitophagy and OXPHOS play synergistic roles in maintaining mitochondrial protein balance and mitochondrial function (Kang et al., 2017; Martinez et al., 2017; Sorrentino et al., 2017; Poole and Macleod, 2021). The latest article published in NATURE refers to a series of mitochondrial stress responses that cause abnormal changes in the UPRmt, mitophagy, and OXPHOS under specific pathological or stimulus conditions, such as the mitochondrial stress response (MSR) (Ruan et al., 2017; Sorrentino et al., 2017; Jakobsen et al., 2019). The MSR is a key mechanism regulating mitochondrial protein balance and the mitochondrial stress response. EPAC1 can activate the UPRmt and protect mitochondrial function. Additionally, it is a key molecule that regulates the UPRmt and mitophagy (Gariani et al., 2016; Jayarajan et al., 2019; Aslam and Ladilov, 2021).

## 10 Summary

TKIs can induce vascular endothelial damage, hypertension and myocardial injury by acting on the targets VEGFR, platelet-derived growth factor receptor (PDGFR) and stem cell factor receptor (SCFR). It can also damage mitochondria and affect myocardial energy metabolism through “off-target effects", eventually leading to cardiovascular complications. Therefore, the goal of antitumour therapy is to maximize the antitumour effect while reducing treatment-related cardiovascular events. Therefore, studying the mechanism of hypertension during the treatment of cancer patients with TKIs and finding compounds or key factors that can reduce blood pressure without affecting antitumour efficacy are highly valuable. We hypothesize that inhibition of Notch signalling could ameliorate TKI-induced microvascular rarefaction, thereby further ameliorating the increase in blood pressure or the resulting cardiac dysfunction caused by microvascular rarefaction. Overexpression of Epac 1, a key gene in the Notch1 signalling pathway, can improve the pathological vascular inhibition induced by TKIs. The mechanism by which apatinib, a representative TKI, induces microcirculation damage, hypertension and heart failure through the Notch1 signalling pathway will also be explored at the cellular and animal levels to provide clinical guidance for patients with hypertension induced by cancer treated with TKIs.

In summary, side effects such as cardiovascular toxicity caused by antitumour therapy with TKIs have become one of the main factors limiting antitumour therapy with TKIs. For the treatment of such hypertension, the effect of traditional antihypertensive drugs is not ideal, and traditional antihypertensive drugs are also closely related to the occurrence and development of some tumours. Exploring the underlying mechanism of cardiovascular complications caused by antitumour treatment with TKIs is crucial for ensuring the smooth clinical application of TKIs, and identifying this mechanism is also an urgent need. New directions for improving the treatment of hypertension induced by these drugs should be explored. The ultimate goal of our team will be to improve the clinical outcome as soon as possible and to provide greater benefits for cancer patients.

## References

[B1] Agabiti-Rosei E. (2003). Structural and functional changes of the microcirculation in hypertension: influence of pharmacological therapy. Drugs 63, 19–29. 12708883

[B2] Agrawal V. Kropski J. A. Gokey J. J. Kobeck E. Murphy M. B. Murray K. T. (2023). Myeloid cell derived IL1β contributes to pulmonary hypertension in HFpEF. Circ. Res. 133 (11), 885–898. 10.1161/CIRCRESAHA.123.323119 37929582 PMC10655859

[B3] Antonios T. F. Rattray F. M. Singer D. R. Markandu N. D. Mortimer P. S. MacGregor G. A. (2003). Rarefaction of skin capillaries in normotensive offspring of individuals with essential hypertension. Heart 89 (2), 175–178. 10.1136/heart.89.2.175 12527671 PMC1767555

[B4] Aslam M. Ladilov Y. (2021). Regulation of mitochondrial homeostasis by sAC-derived cAMP pool: basic and translational aspects. Cells 10 (2), 473. 10.3390/cells10020473 33671810 PMC7926680

[B5] Benedito R. Roca C. Sörensen I. Adams S. Gossler A. Fruttiger M. (2009). The notch ligands Dll4 and Jagged1 have opposing effects on angiogenesis. Cell 137 (6), 1124–1135. 10.1016/j.cell.2009.03.025 19524514

[B6] Bernatz S. Monden D. Gessler F. Radic T. Hattingen E. Senft C. (2021). Influence of VEGF-A, VEGFR-1-3, and neuropilin 1-2 on progression-free: and overall survival in WHO grade II and III meningioma patients. J. Mol. Histol. 52 (2), 233–243. 10.1007/s10735-020-09940-2 33528717 PMC8012320

[B7] Boegehold M. A. Johnson M. D. Overbeck H. W. (1991). Pressure-independent arteriolar rarefaction in hypertension. Am. J. Physiol. 261 (1 Pt 2), H83–H87. 10.1152/ajpheart.1991.261.1.H83 1858934

[B8] Bray F. Laversanne M. Weiderpass E. Soerjomataram I. (2021). The ever-increasing importance of cancer as a leading cause of premature death worldwide. Cancer 127 (16), 3029–3030. 10.1002/cncr.33587 34086348

[B9] Bruno R. M. Masi S. Taddei M. Taddei S. Virdis A. (2018). Essential hypertension and functional microvascular ageing. High. Blood Press Cardiovasc Prev. 25 (1), 35–40. 10.1007/s40292-017-0245-9 29313304

[B10] Camici P. G. Tschöpe C. Di Carli M. F. Rimoldi O. Van Linthout S. (2020). Coronary microvascular dysfunction in hypertrophy and heart failure. Cardiovasc Res. 116 (4), 806–816. 10.1093/cvr/cvaa023 31999329

[B11] Catino A. B. Hubbard R. A. Chirinos J. A. Townsend R. Keefe S. Haas N. B. (2018). Longitudinal assessment of vascular function with sunitinib in patients with metastatic renal cell carcinoma. Circ. Heart Fail 11 (3), e004408. 10.1161/CIRCHEARTFAILURE.117.004408 29664405 PMC6360089

[B12] Chade A. R. Kelsen S. (2010). Renal microvascular disease determines the responses to revascularization in experimental renovascular disease. Circ. Cardiovasc Interv. 3 (4), 376–383. 10.1161/CIRCINTERVENTIONS.110.951277 20587789 PMC3032938

[B13] Chappell J. C. Taylor S. M. Ferrara N. Bautch V. L. (2009). Local guidance of emerging vessel sprouts requires soluble Flt-1. Dev. Cell 17 (3), 377–386. 10.1016/j.devcel.2009.07.011 19758562 PMC2747120

[B14] Ciuffetti G. Schillaci G. Innocente S. Lombardini R. Pasqualini L. Notaristefano S. (2003). Capillary rarefaction and abnormal cardiovascular reactivity in hypertension. J. Hypertens. 21 (12), 2297–2303. 10.1097/00004872-200312000-00018 14654750

[B15] Cohen J. B. Brown N. J. Brown S. A. Dent S. van Dorst D. C. H. Herrmann S. M. (2023). Cancer therapy-related hypertension: a scientific statement from the American heart association. Hypertension 80 (3), e46–e57. 10.1161/HYP.0000000000000224 36621810 PMC10602651

[B16] Cusack R. Leone M. Rodriguez A. H. Martin-Loeches I. (2022). Endothelial damage and the microcirculation in critical illness. Biomedicines 10 (12), 3150. 10.3390/biomedicines10123150 36551905 PMC9776078

[B17] De Backer D. Orbegozo Cortes D. Donadello K. Vincent J. L. (2014). Pathophysiology of microcirculatory dysfunction and the pathogenesis of septic shock. Virulence 5 (1), 73–79. 10.4161/viru.26482 24067428 PMC3916386

[B18] De Ciuceis C. Rizzoni D. Palatini P. (2023). Microcirculation and physical exercise in hypertension. Hypertension 80 (4), 730–739. 10.1161/HYPERTENSIONAHA.122.19465 36601920

[B19] Do T. Van A. Ataei A. Sharma S. Mohandas R. (2023). Microvascular dysfunction in obesity-hypertension. Curr. Hypertens. Rep. 25 (12), 447–453. 10.1007/s11906-023-01272-2 37837517

[B20] Dolmatova E. Waheed N. Olson B. M. Patel S. A. Mandawat A. (2023). The intersection of prostate cancer and hypertension: a call to action. Curr. Treat. Options Oncol. 24 (7), 892–905. 10.1007/s11864-023-01094-z 37191906

[B21] Fang Z. Q. Ruan B. Liu J. J. Duan J. L. Yue Z. S. Song P. (2022). Notch-triggered maladaptation of liver sinusoidal endothelium aggravates nonalcoholic steatohepatitis through endothelial nitric oxide synthase. Hepatology 76 (3), 742–758. 10.1002/hep.32332 35006626

[B22] Fazal L. Laudette M. Paula-Gomes S. Pons S. Conte C. Tortosa F. (2017). Multifunctional mitochondrial Epac1 controls myocardial cell death. Circ. Res. 120 (4), 645–657. 10.1161/CIRCRESAHA.116.309859 28096195

[B23] Feihl F. Liaudet L. Levy B. I. Waeber B. (2008). Hypertension and microvascular remodelling. Cardiovasc Res. 78 (2), 274–285. 10.1093/cvr/cvn022 18250145

[B24] Fortini C. Cesselli D. Beltrami A. P. Bergamin N. Caragnano A. Moretti L. (2014). Alteration of Notch signaling and functionality of adipose tissue derived mesenchymal stem cells in heart failure. Int. J. Cardiol. 174 (1), 119–126. 10.1016/j.ijcard.2014.03.173 24767126

[B25] Fritz A. L. Adil M. M. Mao S. R. Schaffer D. V. (2015). cAMP and EPAC signaling functionally replace OCT4 during induced pluripotent stem cell reprogramming. Mol. Ther. 23 (5), 952–963. 10.1038/mt.2015.28 25666918 PMC4427878

[B26] Fujita T. Umemura M. Yokoyama U. Okumura S. Ishikawa Y. (2017). The role of Epac in the heart. Cell Mol. Life Sci. 74 (4), 591–606. 10.1007/s00018-016-2336-5 27549789 PMC11107744

[B27] Gao Q. He S. Peng Y. Su P. Zhao L. (2023). Proteomic profiling of epicardial fat in heart failure with preserved versus reduced and mildly reduced ejection fraction. J. Cell Mol. Med. 27 (5), 727–735. 10.1111/jcmm.17695 36808702 PMC9983313

[B28] Garg P. Lewis R. A. Johns C. S. Swift A. J. Capener D. Rajaram S. (2021). Cardiovascular magnetic resonance predicts all-cause mortality in pulmonary hypertension associated with heart failure with preserved ejection fraction. Int. J. Cardiovasc Imaging 37 (10), 3019–3025. 10.1007/s10554-021-02279-z 33978936 PMC8494694

[B29] Gariani K. Menzies K. J. Ryu D. Wegner C. J. Wang X. Ropelle E. R. (2016). Eliciting the mitochondrial unfolded protein response by nicotinamide adenine dinucleotide repletion reverses fatty liver disease in mice. Hepatology 63 (4), 1190–1204. 10.1002/hep.28245 26404765 PMC4805450

[B30] Gerhardt H. Golding M. Fruttiger M. Ruhrberg C. Lundkvist A. Abramsson A. (2003). VEGF guides angiogenic sprouting utilizing endothelial tip cell filopodia. J. Cell Biol. 161 (6), 1163–1177. 10.1083/jcb.200302047 12810700 PMC2172999

[B31] Gevaert A. B. Kataria R. Zannad F. Sauer A. J. Damman K. Sharma K. (2022). Heart failure with preserved ejection fraction: recent concepts in diagnosis, mechanisms and management. Heart 108 (17), 1342–1350. 10.1136/heartjnl-2021-319605 35022210

[B32] Giovannini C. Fornari F. Piscaglia F. Gramantieri L. (2021). Notch signaling regulation in HCC: from hepatitis virus to non-coding RNAs. Cells 10 (3), 521. 10.3390/cells10030521 33804511 PMC8000248

[B33] Gomez A. H. Joshi S. Yang Y. Tune J. D. Zhao M. T. Yang H. (2021). Bioengineering systems for modulating notch signaling in cardiovascular development, disease, and regeneration. J. Cardiovasc Dev. Dis. 8 (10), 125. 10.3390/jcdd8100125 34677194 PMC8541010

[B34] Gryglewska B. Nęcki M. Zelawski M. Cwynar M. Baron T. Mrozek M. (2011). Fractal dimensions of skin microcirculation flow in subjects with familial predisposition or newly diagnosed hypertension. Cardiol. J. 18 (1), 26–32. 21305482

[B35] Guo Y. Guan T. Shafiq K. Yu Q. Jiao X. Na D. (2023). Mitochondrial dysfunction in aging. Ageing Res. Rev. 88, 101955. 10.1016/j.arr.2023.101955 37196864

[B36] Herbert S. P. Stainier D. Y. (2011). Molecular control of endothelial cell behaviour during blood vessel morphogenesis. Nat. Rev. Mol. Cell Biol. 12 (9), 551–564. 10.1038/nrm3176 21860391 PMC3319719

[B37] Horton W. B. Barrett E. J. (2021). Microvascular dysfunction in diabetes mellitus and cardiometabolic disease. Endocr. Rev. 42 (1), 29–55. 10.1210/endrev/bnaa025 33125468 PMC7846151

[B38] Hrstka S. C. Li X. Nelson T. J. Wanek Program Genetics Pipeline Group (2017). NOTCH1-Dependent nitric oxide signaling deficiency in hypoplastic left heart syndrome revealed through patient-specific phenotypes detected in bioengineered cardiogenesis. Stem Cells 35 (4), 1106–1119. 10.1002/stem.2582 28142228

[B39] Huang C. Li H. Xu Y. Xu C. Sun H. Li Z. (2023). BICC1 drives pancreatic cancer progression by inducing VEGF-independent angiogenesis. Signal Transduct. Target Ther. 8 (1), 271. 10.1038/s41392-023-01478-5 37443111 PMC10344882

[B40] Jackson A. M. Jhund P. S. Anand I. S. Düngen H. D. Lam C. S. P. Lefkowitz M. P. (2021). Sacubitril-valsartan as a treatment for apparent resistant hypertension in patients with heart failure and preserved ejection fraction. Eur. Heart J. 42 (36), 3741–3752. 10.1093/eurheartj/ehab499 34392331 PMC8455346

[B41] Jacobson A. Yan C. Gao Q. Rincon-Skinner T. Rivera A. Edwards J. (2007). Aging enhances pressure-induced arterial superoxide formation. Am. J. Physiol. Heart Circ. Physiol. 293 (3), H1344–H1350. 10.1152/ajpheart.00413.2007 17557915 PMC4536921

[B42] Jakobsen E. Lange S. C. Bak L. K. (2019). Soluble adenylyl cyclase-mediated cAMP signaling and the putative role of PKA and EPAC in cerebral mitochondrial function. J. Neurosci. Res. 97 (8), 1018–1038. 10.1002/jnr.24477 31172581

[B43] Jakobsson L. Franco C. A. Bentley K. Collins R. T. Ponsioen B. Aspalter I. M. (2010). Endothelial cells dynamically compete for the tip cell position during angiogenic sprouting. Nat. Cell Biol. 12 (10), 943–953. 10.1038/ncb2103 20871601

[B44] Jayarajan V. Appukuttan A. Aslam M. Reusch P. Regitz-Zagrosek V. Ladilov Y. (2019). Regulation of AMPK activity by type 10 adenylyl cyclase: contribution to the mitochondrial biology, cellular redox and energy homeostasis. Cell Mol. Life Sci. 76 (24), 4945–4959. 10.1007/s00018-019-03152-y 31172217 PMC11105217

[B45] Johnson S. E. Barrick D. (2012). Dissecting and circumventing the requirement for RAM in CSL-dependent Notch signaling. PLoS One 7 (8), e39093. 10.1371/journal.pone.0039093 22876274 PMC3410904

[B46] Jonk A. M. Houben A. J. de Jongh R. T. Serné E. H. Schaper N. C. Stehouwer C. D. (2007). Microvascular dysfunction in obesity: a potential mechanism in the pathogenesis of obesity-associated insulin resistance and hypertension. Physiol. (Bethesda) 22, 252–260. 10.1152/physiol.00012.2007 17699878

[B47] Kang M. H. Das J. Gurunathan S. Park H. W. Song H. Park C. (2017). The cytotoxic effects of dimethyl sulfoxide in mouse preimplantation embryos: a mechanistic study. Theranostics 7 (19), 4735–4752. 10.7150/thno.21662 29187900 PMC5706096

[B48] Kangsamaksin T. Murtomaki A. Kofler N. M. Cuervo H. Chaudhri R. A. Tattersall I. W. (2015). NOTCH decoys that selectively block DLL/NOTCH or JAG/NOTCH disrupt angiogenesis by unique mechanisms to inhibit tumor growth. Cancer Discov. 5 (2), 182–197. 10.1158/2159-8290.CD-14-0650 25387766 PMC4423829

[B49] Karaca Ü. Schram M. T. Houben A. J. Muris D. M. Stehouwer C. D. (2014). Microvascular dysfunction as a link between obesity, insulin resistance and hypertension. Diabetes Res. Clin. Pract. 103 (3), 382–387. 10.1016/j.diabres.2013.12.012 24438874

[B50] Kumar R. Chaudhary A. K. Woytash J. Inigo J. R. Gokhale A. A. Bshara W. (2022). A mitochondrial unfolded protein response inhibitor suppresses prostate cancer growth in mice via HSP60. J. Clin. Invest. 132 (13), e149906. 10.1172/JCI149906 35653190 PMC9246382

[B51] Kwak M. Southard K. M. Kim W. R. Lin A. Kim N. H. Gopalappa R. (2022). Adherens junctions organize size-selective proteolytic hotspots critical for Notch signalling. Nat. Cell Biol. 24 (12), 1739–1753. 10.1038/s41556-022-01031-6 36456828 PMC10665132

[B52] Laham R. J. Li J. Tofukuji M. Post M. Simons M. Sellke F. W. (2003). Spatial heterogeneity in VEGF-induced vasodilation: VEGF dilates microvessels but not epicardial and systemic arteries and veins. Ann. Vasc. Surg. 17 (3), 245–252. 10.1007/s10016-001-0299-x 12704544

[B53] Lan C. Shen J. Wang Y. Li J. Liu Z. He M. (2020). Camrelizumab plus apatinib in patients with advanced cervical cancer (clap): a multicenter, open-label, single-arm, phase II trial. J. Clin. Oncol. 38 (34), 4095–4106. 10.1200/JCO.20.01920 33052760 PMC7768345

[B54] Laudette M. Sainte-Marie Y. Cousin G. Bergonnier D. Belhabib I. Brun S. (2021). Cyclic AMP-binding protein Epac1 acts as a metabolic sensor to promote cardiomyocyte lipotoxicity. Cell Death Dis. 12 (9), 824. 10.1038/s41419-021-04113-9 34471096 PMC8410846

[B55] Lawler J. (2022). Counter regulation of tumor angiogenesis by vascular endothelial growth factor and thrombospondin-1. Semin. Cancer Biol. 86 (Pt 2), 126–135. 10.1016/j.semcancer.2022.09.006 36191900

[B56] Le X. Nilsson M. Goldman J. Reck M. Nakagawa K. Kato T. (2021). Dual EGFR-VEGF pathway inhibition: a promising strategy for patients with EGFR-mutant nsclc. J. Thorac. Oncol. 16 (2), 205–215. 10.1016/j.jtho.2020.10.006 33096270

[B57] Lennartsson J. Rönnstrand L. (2012). Stem cell factor receptor/c-Kit: from basic science to clinical implications. Physiol. Rev. 92 (4), 1619–1649. 10.1152/physrev.00046.2011 23073628

[B58] le Noble F. A. C. Mourad J. J. Levy B. I. Struijker-Boudier H. A. J. (2023). VEGF (vascular endothelial growth factor) inhibition and hypertension: does microvascular rarefaction play a role? Hypertension 80 (5), 901–911. 10.1161/HYPERTENSIONAHA.122.19427 36748474

[B59] Levy B. I. Ambrosio G. Pries A. R. Struijker-Boudier H. A. (2001). Microcirculation in hypertension: a new target for treatment? Circulation 104 (6), 735–740. 10.1161/hc3101.091158 11489784

[B60] Levy B. I. Schiffrin E. L. Mourad J. J. Agostini D. Vicaut E. Safar M. E. (2008). Impaired tissue perfusion: a pathology common to hypertension, obesity, and diabetes mellitus. Circulation 118 (9), 968–976. 10.1161/CIRCULATIONAHA.107.763730 18725503

[B61] Li C. Ma L. Wang Q. Shao X. Guo L. Chen J. (2022). Rho kinase inhibition ameliorates vascular remodeling and blood pressure elevations in a rat model of apatinib-induced hypertension. J. Hypertens. 40 (4), 675–684. 10.1097/HJH.0000000000003060 34862331 PMC8901036

[B62] Li J. Qin S. Xu J. Xiong J. Wu C. Bai Y. (2016). Randomized, double-blind, placebo-controlled phase III trial of apatinib in patients with chemotherapy-refractory advanced or metastatic adenocarcinoma of the stomach or gastroesophageal junction. J. Clin. Oncol. 34 (13), 1448–1454. 10.1200/JCO.2015.63.5995 26884585

[B63] Li L. Liu H. Xu C. Deng M. Song M. Yu X. (2017). VEGF promotes endothelial progenitor cell differentiation and vascular repair through connexin 43. Stem Cell Res. Ther. 8 (1), 237. 10.1186/s13287-017-0684-1 29065929 PMC5655878

[B64] Li S. Shi Y. Yuan S. Ruan J. Pan H. Ma M. (2024). Inhibiting the MAPK pathway improves heart failure with preserved ejection fraction induced by salt-sensitive hypertension. Biomed. Pharmacother. 170, 115987. 10.1016/j.biopha.2023.115987 38056241

[B65] Li T. Xu X. H. Guo X. Yuan T. Tang Z. H. Jiang X. M. (2020). Activation of notch 3/c-MYC/CHOP axis regulates apoptosis and promotes sensitivity of lung cancer cells to mTOR inhibitor everolimus. Biochem. Pharmacol. 175, 113921. 10.1016/j.bcp.2020.113921 32201213

[B66] Li X. Souilhol C. Canham L. Jia X. Diagbouga M. Ayllon B. T. (2023a). DLL4 promotes partial endothelial-to-mesenchymal transition at atherosclerosis-prone regions of arteries. Vasc. Pharmacol. 150, 107178. 10.1016/j.vph.2023.107178 37137436

[B67] Li X. Yan X. Wang Y. Kaur B. Han H. Yu J. (2023b). The Notch signaling pathway: a potential target for cancer immunotherapy. J. Hematol. Oncol. 16 (1), 45. 10.1186/s13045-023-01439-z 37131214 PMC10155406

[B68] Lubman O. Y. Ilagan M. X. Kopan R. Barrick D. (2007). Quantitative dissection of the Notch:CSL interaction: insights into the Notch-mediated transcriptional switch. J. Mol. Biol. 365 (3), 577–589. 10.1016/j.jmb.2006.09.071 17070841 PMC1851696

[B69] MacGrogan D. Münch J. de la Pompa J. L. (2018). Notch and interacting signalling pathways in cardiac development, disease, and regeneration. Nat. Rev. Cardiol. 15 (11), 685–704. 10.1038/s41569-018-0100-2 30287945

[B70] Marinescu M. A. Löffler A. I. Ouellette M. Smith L. Kramer C. M. Bourque J. M. (2015). Coronary microvascular dysfunction, microvascular angina, and treatment strategies. JACC Cardiovasc Imaging 8 (2), 210–220. 10.1016/j.jcmg.2014.12.008 25677893 PMC4384521

[B71] Marra A. M. Sherman A. E. Salzano A. Guazzi M. Saggar R. Squire I. B. (2022). Right side of the heart pulmonary circulation unit involvement in left-sided heart failure: diagnostic, prognostic, and therapeutic implications. Chest 161 (2), 535–551. 10.1016/j.chest.2021.09.023 34592320

[B72] Martens J. R. Gelband C. H. (1998). Ion channels in vascular smooth muscle: alterations in essential hypertension. Proc. Soc. Exp. Biol. Med. 218 (3), 192–203. 10.3181/00379727-218-44286 9648936

[B73] Martinez B. A. Petersen D. A. Gaeta A. L. Stanley S. P. Caldwell G. A. Caldwell K. A. (2017). Dysregulation of the mitochondrial unfolded protein response induces non-apoptotic dopaminergic neurodegeneration in *C. elegans* models of Parkinson's disease. J. Neurosci. 37 (46), 11085–11100. 10.1523/JNEUROSCI.1294-17.2017 29030433 PMC5688521

[B74] Matsushita K. Marchandot B. Trimaille A. Hmadeh S. Kibler M. Heger J. (2023). Determinants and treatments of heart failure after transcatheter aortic valve implantation: moving up a notch. Esc. Heart Fail 10 (4), 2183–2199. 10.1002/ehf2.14435 37430483 PMC10375170

[B75] Maynard S. E. Min J. Y. Merchan J. Lim K. H. Li J. Mondal S. (2003). Excess placental soluble fms-like tyrosine kinase 1 (sFlt1) may contribute to endothelial dysfunction, hypertension, and proteinuria in preeclampsia. J. Clin. Invest. 111 (5), 649–658. 10.1172/JCI17189 12618519 PMC151901

[B76] Mengozzi A. de Ciuceis C. Dell'oro R. Georgiopoulos G. Lazaridis A. Nosalski R. (2023). The importance of microvascular inflammation in ageing and age-related diseases: a position paper from the ESH working group on small arteries, section of microvascular inflammation. J. Hypertens. 41 (10), 1521–1543. 10.1097/HJH.0000000000003503 37382158

[B77] Merkwirth C. Jovaisaite V. Durieux J. Matilainen O. Jordan S. D. Quiros P. M. (2016). Two conserved histone demethylases regulate mitochondrial stress-induced longevity. Cell 165 (5), 1209–1223. 10.1016/j.cell.2016.04.012 27133168 PMC4889222

[B78] Milan A. Puglisi E. Ferrari L. Bruno G. Losano I. Veglio F. (2014). Arterial hypertension and cancer. Int. J. Cancer 134 (10), 2269–2277. 10.1002/ijc.28334 23784914

[B79] Mirabito Colafella K. M. Neves K. B. Montezano A. C. Garrelds I. M. van Veghel R. de Vries R. (2020). Selective ETA vs dual ETA/B receptor blockade for the prevention of sunitinib-induced hypertension and albuminuria in WKY rats. Cardiovasc. Res. 116 (10), 1779–1790. 10.1093/cvr/cvz260 31593221

[B80] Mourad J. J. des Guetz G. Debbabi H. Levy B. I. (2008). Blood pressure rise following angiogenesis inhibition by bevacizumab. A crucial role for microcirculation. Ann. Oncol. 19 (5), 927–934. 10.1093/annonc/mdm550 18056916

[B81] Mourad J. J. Levy B. I. (2011). Mechanisms of antiangiogenic-induced arterial hypertension. Curr. Hypertens. Rep. 13 (4), 289–293. 10.1007/s11906-011-0206-y 21479992

[B82] Musheshe N. Oun A. Sabogal-Guáqueta A. M. Trombetta-Lima M. Mitchel S. C. Adzemovic A. (2022). Pharmacological inhibition of Epac1 averts ferroptosis cell death by preserving mitochondrial integrity. Antioxidants (Basel) 11 (2), 314. 10.3390/antiox11020314 35204198 PMC8868285

[B83] Nagai M. Dote K. Förster C. Y. (2023). Denervation or stimulation? Role of sympatho-vagal imbalance in HFpEF with hypertension. Hypertens. Res. 46 (7), 1727–1737. 10.1038/s41440-023-01272-4 37045971

[B84] Namkoong S. Kim C. K. Cho Y. L. Kim J. H. Lee H. Ha K. S. (2009). Forskolin increases angiogenesis through the coordinated cross-talk of PKA-dependent VEGF expression and Epac-mediated PI3K/Akt/eNOS signaling. Cell Signal 21 (6), 906–915. 10.1016/j.cellsig.2009.01.038 19385062

[B85] Neves K. B. Rios F. J. Jones R. Evans T. R. J. Montezano A. C. Touyz R. M. (2019). Microparticles from vascular endothelial growth factor pathway inhibitor-treated cancer patients mediate endothelial cell injury. Cardiovasc Res. 115 (5), 978–988. 10.1093/cvr/cvz021 30753341 PMC6452312

[B86] Neves K. B. Rios F. J. van der Mey L. Alves-Lopes R. Cameron A. C. Volpe M. (2018). VEGFR (vascular endothelial growth factor receptor) inhibition induces cardiovascular damage via redox-sensitive processes. Hypertension 71 (4), 638–647. 10.1161/HYPERTENSIONAHA.117.10490 29483228

[B87] Niessen K. Karsan A. (2007). Notch signaling in the developing cardiovascular system. Am. J. Physiol. Cell Physiol. 293 (1), C1–C11. 10.1152/ajpcell.00415.2006 17376817

[B88] Niessen K. Zhang G. Ridgway J. B. Chen H. Kolumam G. Siebel C. W. (2011). The Notch1-Dll4 signaling pathway regulates mouse postnatal lymphatic development. Blood 118 (7), 1989–1997. 10.1182/blood-2010-11-319129 21700774

[B89] Obokata M. Reddy Y. N. V. Pislaru S. V. Melenovsky V. Borlaug B. A. (2017). Evidence supporting the existence of a distinct obese phenotype of heart failure with preserved ejection fraction. Circulation 136 (1), 6–19. 10.1161/CIRCULATIONAHA.116.026807 28381470 PMC5501170

[B90] Pahlavani H. A. (2022). Exercise-induced signaling pathways to counteracting cardiac apoptotic processes. Front. Cell Dev. Biol. 10, 950927. 10.3389/fcell.2022.950927 36036015 PMC9403089

[B91] Paulus W. J. Tschöpe C. (2013). A novel paradigm for heart failure with preserved ejection fraction: comorbidities drive myocardial dysfunction and remodeling through coronary microvascular endothelial inflammation. J. Am. Coll. Cardiol. 62 (4), 263–271. 10.1016/j.jacc.2013.02.092 23684677

[B92] Peng X. Wang S. Chen H. Chen M. (2023). Role of the Notch1 signaling pathway in ischemic heart disease (Review). Int. J. Mol. Med. (3), 51. 10.3892/ijmm.2023.5230 36799152

[B93] Poole L. P. Macleod K. F. (2021). Mitophagy in tumorigenesis and metastasis. Cell Mol. Life Sci. 78 (8), 3817–3851. 10.1007/s00018-021-03774-1 33580835 PMC8259496

[B94] Poznyak A. V. Nikiforov N. G. Wu W. K. Kirichenko T. V. Orekhov A. N. (2021). Autophagy and mitophagy as essential components of atherosclerosis. Cells 10 (2), 443. 10.3390/cells10020443 33669743 PMC7922388

[B95] Premont R. T. Reynolds J. D. Zhang R. Stamler J. S. (2020). Role of nitric oxide carried by hemoglobin in cardiovascular physiology: developments on a three-gas respiratory cycle. Circ. Res. 126 (1), 129–158. 10.1161/CIRCRESAHA.119.315626 31590598 PMC7034631

[B96] Pucci G. Milan A. Paini A. Salvetti M. Cerasari A. Vaudo G. (2019). Acute blood pressure elevation associated with biological therapies for cancer: a focus on VEGF signaling pathway inhibitors. Expert Opin. Biol. Ther. 19 (5), 433–442. 10.1080/14712598.2019.1594770 30888868

[B97] Qin S. Chan S. L. Gu S. Bai Y. Ren Z. Lin X. (2023). Camrelizumab plus rivoceranib versus sorafenib as first-line therapy for unresectable hepatocellular carcinoma (CARES-310): a randomised, open-label, international phase 3 study. Lancet 402 (10408), 1133–1146. 10.1016/S0140-6736(23)00961-3 37499670

[B98] Ramos C. J. Lin C. Liu X. Antonetti D. A. (2018). The EPAC-Rap1 pathway prevents and reverses cytokine-induced retinal vascular permeability. J. Biol. Chem. 293 (2), 717–730. 10.1074/jbc.M117.815381 29158262 PMC5767874

[B99] Redfield M. M. Borlaug B. A. (2023). Heart failure with preserved ejection fraction: a review. Jama 329 (10), 827–838. 10.1001/jama.2023.2020 36917048

[B100] Rizzoni D. Agabiti-Rosei C. Boari G. E. M. Muiesan M. L. De Ciuceis C. (2023). Microcirculation in hypertension: a therapeutic target to prevent cardiovascular disease? J. Clin. Med. 12 (15), 4892. 10.3390/jcm12154892 37568294 PMC10419740

[B101] Robinson E. S. Khankin E. V. Choueiri T. K. Dhawan M. S. Rogers M. J. Karumanchi S. A. (2010). Suppression of the nitric oxide pathway in metastatic renal cell carcinoma patients receiving vascular endothelial growth factor-signaling inhibitors. Hypertension 56 (6), 1131–1136. 10.1161/HYPERTENSIONAHA.110.160481 20956731 PMC3078049

[B102] Rodríguez-Agustín A. Casanova V. Grau-Expósito J. Sánchez-Palomino S. Alcamí J. Climent N. (2023). Immunomodulatory activity of the tyrosine kinase inhibitor dasatinib to elicit NK cytotoxicity against cancer, HIV infection and aging. Pharmaceutics 15 (3), 917. 10.3390/pharmaceutics15030917 36986778 PMC10055786

[B103] Rossi V. A. Nebunu D. Haider T. Laptseva N. Naegele M. P. Ruschitzka F. (2023). Diverging role of epicardial adipose tissue across the entire heart failure spectrum. Esc. Heart Fail 10 (6), 3419–3429. 10.1002/ehf2.14483 37697706 PMC10682858

[B104] Ruan L. Zhou C. Jin E. Kucharavy A. Zhang Y. Wen Z. (2017). Cytosolic proteostasis through importing of misfolded proteins into mitochondria. Nature 543 (7645), 443–446. 10.1038/nature21695 28241148 PMC5793917

[B105] Sabe S. A. Feng J. Sellke F. W. Abid M. R. (2022). Mechanisms and clinical implications of endothelium-dependent vasomotor dysfunction in coronary microvasculature. Am. J. Physiol. Heart Circ. Physiol. 322 (5), H819–h841. 10.1152/ajpheart.00603.2021 35333122 PMC9018047

[B106] Saif M. W. (2013). Anti-VEGF agents in metastatic colorectal cancer (mCRC): are they all alike? Cancer Manag. Res. 5, 103–115. 10.2147/CMAR.S45193 23807861 PMC3685399

[B107] Sallinen H. Anttila M. Narvainen J. Koponen J. Hamalainen K. Kholova I. (2009). Antiangiogenic gene therapy with soluble VEGFR-1, -2, and -3 reduces the growth of solid human ovarian carcinoma in mice. Mol. Ther. 17 (2), 278–284. 10.1038/mt.2008.258 19050699 PMC2835051

[B108] Serné E. H. Gans R. O. ter Maaten J. C. Tangelder G. J. Donker A. J. Stehouwer C. D. (2001). Impaired skin capillary recruitment in essential hypertension is caused by both functional and structural capillary rarefaction. Hypertension 38 (2), 238–242. 10.1161/01.hyp.38.2.238 11509483

[B109] Shughoury A. Bhatwadekar A. Jusufbegovic D. Hajrasouliha A. Ciulla T. A. (2023). The evolving therapeutic landscape of diabetic retinopathy. Expert Opin. Biol. Ther. 23 (10), 969–985. 10.1080/14712598.2023.2247987 37578843 PMC10592121

[B110] Slika H. Mansour H. Nasser S. A. Shaito A. Kobeissy F. Orekhov A. N. (2023). Epac as a tractable therapeutic target. Eur. J. Pharmacol. 945, 175645. 10.1016/j.ejphar.2023.175645 36894048

[B111] Slivnick J. Lampert B. C. (2019). Hypertension and heart failure. Heart Fail Clin. 15 (4), 531–541. 10.1016/j.hfc.2019.06.007 31472888

[B112] Sorop O. van de Wouw J. Chandler S. Ohanyan V. Tune J. D. Chilian W. M. (2020). Experimental animal models of coronary microvascular dysfunction. Cardiovasc Res. 116 (4), 756–770. 10.1093/cvr/cvaa002 31926020 PMC7061277

[B113] Sorrentino V. Romani M. Mouchiroud L. Beck J. S. Zhang H. D'Amico D. (2017). Enhancing mitochondrial proteostasis reduces amyloid-β proteotoxicity. Nature 552 (7684), 187–193. 10.1038/nature25143 29211722 PMC5730497

[B114] Souilhol C. Tardajos Ayllon B. Li X. Diagbouga M. R. Zhou Z. Canham L. (2022). JAG1-NOTCH4 mechanosensing drives atherosclerosis. Sci. Adv. 8 (35), eabo7958. 10.1126/sciadv.abo7958 36044575 PMC9432841

[B115] Sprinzak D. Blacklow S. C. (2021). Biophysics of notch signaling. Annu. Rev. Biophys. 50, 157–189. 10.1146/annurev-biophys-101920-082204 33534608 PMC8105286

[B116] Steeghs N. Gelderblom H. Roodt J. O. Christensen O. Rajagopalan P. Hovens M. (2008). Hypertension and rarefaction during treatment with telatinib, a small molecule angiogenesis inhibitor. Clin. Cancer Res. 14 (11), 3470–3476. 10.1158/1078-0432.CCR-07-5050 18519779

[B117] Struijker-Boudier H. A. Heijnen B. F. (2011). Early life microcirculation and the development of hypertension. Hypertension 58 (5), 768–769. 10.1161/HYPERTENSIONAHA.111.181107 21968758

[B118] Stupin A. Drenjančević I. Šušnjara P. Debeljak Ž. Kolobarić N. Jukić I. (2021). Is there association between altered adrenergic system activity and microvascular endothelial dysfunction induced by a 7-day high salt intake in young healthy individuals. Nutrients 13 (5), 1731. 10.3390/nu13051731 34065261 PMC8161165

[B119] Takahashi H. Sakakibara-Konishi J. Furuta M. Shoji T. Tsuji K. Morinaga D. (2023). Notch pathway regulates osimertinib drug-tolerant persistence in EGFR-mutated non-small-cell lung cancer. Cancer Sci. 114 (4), 1635–1650. 10.1111/cas.15674 36411521 PMC10067397

[B120] Tan Y. Q. Li J. Chen H. W. (2022). Epac, a positive or negative signaling molecule in cardiovascular diseases. Biomed. Pharmacother. 148, 112726. 10.1016/j.biopha.2022.112726 35183995

[B121] Triantafyllou A. Anyfanti P. Pyrpasopoulou A. Triantafyllou G. Aslanidis S. Douma S. (2015). Capillary rarefaction as an index for the microvascular assessment of hypertensive patients. Curr. Hypertens. Rep. 17 (5), 33. 10.1007/s11906-015-0543-3 25833455

[B122] Trindade A. Djokovic D. Gigante J. Badenes M. Pedrosa A. R. Fernandes A. C. (2012). Low-dosage inhibition of Dll4 signaling promotes wound healing by inducing functional neo-angiogenesis. PLoS One 7 (1), e29863. 10.1371/journal.pone.0029863 22279550 PMC3261161

[B123] Tsai S. H. Lu G. Xu X. Ren Y. Hein T. W. Kuo L. (2017). Enhanced endothelin-1/Rho-kinase signalling and coronary microvascular dysfunction in hypertensive myocardial hypertrophy. Cardiovasc Res. 113 (11), 1329–1337. 10.1093/cvr/cvx103 28575410 PMC5852513

[B124] Tullemans B. M. E. Heemskerk J. W. M. Kuijpers M. J. E. (2018). Acquired platelet antagonism: off-target antiplatelet effects of malignancy treatment with tyrosine kinase inhibitors. J. Thromb. Haemost. 16 (9), 1686–1699. 10.1111/jth.14225 29975003

[B125] Ungvari Z. Csiszar A. Kaminski P. M. Wolin M. S. Koller A. (2004). Chronic high pressure-induced arterial oxidative stress: involvement of protein kinase C-dependent NAD(P)H oxidase and local renin-angiotensin system. Am. J. Pathol. 165 (1), 219–226. 10.1016/S0002-9440(10)63290-7 15215177 PMC1618527

[B126] Ungvari Z. Toth P. Tarantini S. Prodan C. I. Sorond F. Merkely B. (2021). Hypertension-induced cognitive impairment: from pathophysiology to public health. Nat. Rev. Nephrol. 17 (10), 639–654. 10.1038/s41581-021-00430-6 34127835 PMC8202227

[B127] Vancheri F. Longo G. Vancheri S. Henein M. (2020). Coronary microvascular dysfunction. J. Clin. Med. 9 (9), 2880. 10.3390/jcm9092880 32899944 PMC7563453

[B128] van Cruijsen H. van der Veldt A. Hoekman K. (2009). Tyrosine kinase inhibitors of VEGF receptors: clinical issues and remaining questions. Front. Biosci. Landmark Ed. 14 (6), 2248–2268. 10.2741/3377 19273199

[B129] van Dorst D. C. H. Dobbin S. J. H. Neves K. B. Herrmann J. Herrmann S. M. Versmissen J. (2021). Hypertension and prohypertensive antineoplastic therapies in cancer patients. Circ. Res. 128 (7), 1040–1061. 10.1161/CIRCRESAHA.121.318051 33793337 PMC8011349

[B130] Vano Y. A. Elaidi R. Bennamoun M. Chevreau C. Borchiellini D. Pannier D. (2022). Nivolumab, nivolumab-ipilimumab, and VEGFR-tyrosine kinase inhibitors as first-line treatment for metastatic clear-cell renal cell carcinoma (BIONIKK): a biomarker-driven, open-label, non-comparative, randomised, phase 2 trial. Lancet Oncol. 23 (5), 612–624. 10.1016/S1470-2045(22)00128-0 35390339

[B131] Viallard C. Larrivée B. (2017). Tumor angiogenesis and vascular normalization: alternative therapeutic targets. Angiogenesis 20 (4), 409–426. 10.1007/s10456-017-9562-9 28660302

[B132] Vimalraj S. (2022). A concise review of VEGF, PDGF, FGF, Notch, angiopoietin, and HGF signalling in tumor angiogenesis with a focus on alternative approaches and future directions. Int. J. Biol. Macromol. 221, 1428–1438. 10.1016/j.ijbiomac.2022.09.129 36122781

[B133] Wang S. Zhu G. Jiang D. Rhen J. Li X. Liu H. (2022c). Reduced Notch1 cleavage promotes the development of pulmonary hypertension. Hypertension 79 (1), 79–92. 10.1161/HYPERTENSIONAHA.120.16065 34739767 PMC8665100

[B134] Wang W. He Q. Li C. Zhuang C. Zhang H. Wang Q. (2022a). Research on the mechanism and prevention of hypertension caused by apatinib through the RhoA/ROCK signaling pathway in a mouse model of gastric cancer. Front. Cardiovasc Med. 9, 873829. 10.3389/fcvm.2022.873829 35811723 PMC9262125

[B135] Wang W. He Q. Zhang H. Zhuang C. Wang Q. Li C. (2021). A narrative review on the interaction between genes and the treatment of hypertension and breast cancer. Ann. Transl. Med. 9 (10), 894. 10.21037/atm-21-2133 34164528 PMC8184430

[B136] Wang W. He Q. Zhuang C. Zhang H. Fan X. Wang Q. (2022b). Apatinib through activating the RhoA/ROCK signaling pathway to cause dysfunction of vascular smooth muscle cells. Appl. Biochem. Biotechnol. 194 (11), 5367–5385. 10.1007/s12010-022-04020-5 35776338

[B137] Wheeler-Jones C. Abu-Ghazaleh R. Cospedal R. Houliston R. A. Martin J. Zachary I. (1997). Vascular endothelial growth factor stimulates prostacyclin production and activation of cytosolic phospholipase A2 in endothelial cells via p42/p44 mitogen-activated protein kinase. FEBS Lett. 420 (1), 28–32. 10.1016/s0014-5793(97)01481-6 9450544

[B138] Wu Y. Fu J. Huang Y. Duan R. Zhang W. Wang C. (2023). Biology and function of pericytes in the vascular microcirculation. Anim. Model Exp. Med. 6 (4), 337–345. 10.1002/ame2.12334 PMC10486323 37317664

[B139] Wu Y. Jiang T. Hua J. Xiong Z. Dai K. Chen H. (2022). PINK1/Parkin-mediated mitophagy in cardiovascular disease: from pathogenesis to novel therapy. Int. J. Cardiol. 361, 61–69. 10.1016/j.ijcard.2022.05.025 35594994

[B140] Xin N. Durieux J. Yang C. Wolff S. Kim H. E. Dillin A. (2022). The UPRmt preserves mitochondrial import to extend lifespan. J. Cell Biol. 221 (7), 221. 10.1083/jcb.202201071 PMC9134095 35608535

[B141] Xu J. Shen J. Gu S. Zhang Y. Wu L. Wu J. (2021). Camrelizumab in combination with apatinib in patients with advanced hepatocellular carcinoma (rescue): a nonrandomized, open-label, phase II trial. Clin. Cancer Res. 27 (4), 1003–1011. 10.1158/1078-0432.CCR-20-2571 33087333

[B142] Yang Z. Qi Y. Lai N. Zhang J. Chen Z. Liu M. (2018). Notch1 signaling in melanoma cells promoted tumor-induced immunosuppression via upregulation of TGF-β1. J. Exp. Clin. Cancer Res. 37 (1), 1. 10.1186/s13046-017-0664-4 29301578 PMC5755139

[B143] Zdravkovic M. Popadic V. Klasnja S. Klasnja A. Ivankovic T. Lasica R. (2023). Coronary microvascular dysfunction and hypertension: a bond more important than we think. Med. Kaunas. 59 (12), 2149. 10.3390/medicina59122149 PMC10744540 38138252

[B144] Zhao L. Ben-Yair R. Burns C. E. Burns C. G. (2019). Endocardial notch signaling promotes cardiomyocyte proliferation in the regenerating zebrafish heart through wnt pathway antagonism. Cell Rep. 26 (3), 546–554. 10.1016/j.celrep.2018.12.048 30650349 PMC6366857

[B145] Zhao Y. Wang Q. Zhao X. Meng H. Yu J. (2018). Effect of antihypertensive drugs on breast cancer risk in female hypertensive patients: evidence from observational studies. Clin. Exp. Hypertens. 40 (1), 22–27. 10.1080/10641963.2017.1288736 29115847

[B146] Zhou B. Lin W. Long Y. Yang Y. Zhang H. Wu K. (2022). Notch signaling pathway: architecture, disease, and therapeutics. Signal Transduct. Target Ther. 7 (1), 95. 10.1038/s41392-022-00934-y 35332121 PMC8948217

